# Medical Items

**Published:** 1898-02

**Authors:** 


### MEDICAL ITEMS.

*Subscribers sometimes complain about not receiving their Journals. We hope all will notify us every time their Journals are not delivered.*

---

Dr. J. C. Drake, of Thomaston, Georgia, died January 8th, in the eighty-fourth year of his age.

---

Dr. Ernest Hart, the learned and distinguished editor of the *British Medical Journal,* died January 7th.

---

Dr. Michael Hoke has been appointed local surgeon to the Seaboard Railroad at Atlanta, succeeding Dr. P. M. Butler.

---

Dr. Joseph O'Dwyer, of New York, inventor of the intubation tubes which bear his name, died January 7th, of tubercular meningitis.

---

Drs. Chas. F. Benson and Louis H. Jones have been elected members of the Atlanta Board of Health, in place of Drs. H. P. Cooper and F. W. McRae, resigned.

---

Officers of the Columbus (Georgia) Academy of Medicine for 1898: President, Dr. George J. Grimes; Vice-President, Dr. T. E. Mitchell; Secretary and Treasurer, Dr. Chas. D. Wall.

---

The New York State Board of Health have so far realized the importance of ocular hygiene that a consulting ophthalmologist has been appointed. Dr. P. A. Callan, of New York City, has been appointed to this position.

DIED.—DR. W. C. NIXON near Rome, Georgia, January 13th, aged fifty-five years. Dr. T. N. Bigham, of Madison, Georgia, January 14th. Dr. John S. Naramore, of Blakely, Georgia, January 22d. Dr. Naramore graduated at the Atlanta Medical College last April. Dr. A. W. Purifoy, of Hawkinsville, aged sixty-nine years. Dr. James F. Barron, of Clinton. January 18th, aged seventy.

---

WHAT do these men all over the country do with the sample copies they write for? Is it a way of keeping cognizant of current medical literature? Judging from the frequency of requests, the amount paid for postage, if judiciously expended, would buy several good journals.—*Atlantic Med. Weekly.*

---

DR. AND MRS. CHAS. E. J. SMITH have opened a Sanitorium, at No. 20 Church street, this city, for the use of special treatments of various diseased conditions, as indicated on another page. Knowing Dr. and Mrs. Smith well, we can unreservedly recommend their institution to physicians and their patients.

---

IN our last issue an error was made in naming the officers of Medical Board of the Grady Hospital for 1898. At the annual meeting of the Board, January 11th, the following officers were elected for the coming year: President, Dr. Jas. B. Baird; Vice-President, Dr. Virgil O. Hardon; Secretary, Dr. Dunbar Roy.

---

"ON New Year's day Mobile honored herself," says the *Mobile Register*, "by honoring the President of the Board of Health, Dr. Geo. A. Ketchum." A reception was held in honor of Dr. Ketchum at the Manassas Club, given by the "Can't-Get-Away Club." Dr. Ketchum was presented with a magnificent sterling silver fruit set.

PROFESSOR TARNIER, of Paris, died recently in that city at the age of seventy years. He was graduated in medicine in 1857 from the University of Paris, and held the chair of clinical obstetrics in that university at the time of his death. He was well known for his many works on obstetrics. Tarnier's forceps are known to every obstetrician.

---

DR. T. C. JOHNSON, of Richland, has obtained a verdict of $16,000 damages against the Central Railroad of Georgia. Several months ago Dr. Johnson was severely injured in a wreck on the Central road, in consequence of which his health and capacity for work have been seriously and permanently impaired. The verdict is one of the largest that has been awarded in Georgia courts.

---

THE fourth annual meeting of the American Laryngological, Rhinological and Otological Society will be held in Pittsburg, Pa., on May 11th and 12th, 1898, under the presidency of Dr. W. H. Daly, of that city. This is the general meeting of the whole society. The Southern Section of this society will convene in Atlanta on March 28, 1898, under the leadership of its chairman, Dr. A. W. Calhoun.

---

DR. KARL H. VON KLEIN has opened a bureau of medical literature at No. 248 E. Erie street, Chicago, to furnish medical literature to medical writers on every subject from its earliest history and from every language in which medicine is written. Dr. von Klein is well known as one of the most fluent linguists on both sides of the Atlantic. This bureau will be a great help to busy physicians, or to physicians who do not have access to large medical libraries.

---

AT the recent annual meeting of the New York Obstetrical Society the following officers were elected for the ensuing year: President, Dr. W. Gill Wylie; Vice-Presidents, Dr. J. C. Edgar and

Dr. A. M. Jacobus; Recording Secretary, Dr. Le Roy Braun; Assistant Recording Secretary, Dr. Geo. W. Jarman; Corresponding Secretary, Dr. E. B. Cragin; Treasurer, Dr. J. Lee Morrill; Pathologist, Dr. Geo. C. Freeborn.

---

THE SENN MEDAL.—Those members of the American Medical Association intending to compete for the medal offered by Dr. Senn for the best essay on some surgical subject, are requested to forward the type-written copies of contributions before March 1, 1898. Address J. McFadden Gaston, M.D., Chm., Atlanta, Ga.

---

WE have received the transactions of the American Orthopedic Association for the eleventh annual meeting in Washington, May 4th to 6th, 1897. The volume is well bound, containing 260 pages devoted to papers upon orthopedic surgery. The officers for the current year are: Dr. R. W. Lovitt, President; Drs. W. R. Townsend and J. E. Goldthwait, Vice-Presidents; Dr. E. G. Brockett, Treasurer; Dr. John Ridlon (103 State St., Chicago), Secretary.

---

THE Virginia Hospital and the Richmond (Va.) Male Orphan Asylum will each receive $10,000 from the estate of the late Major Lewis Ginter; St. Luke's Home for the Sick, the Sheltering Arms Hospital, and Richmond Eye, Ear and Throat Infirmary each receive $5,000, while $2,500 will go to the Maternity Hospital and $1,000 to the Foundling Hospital. Richmond owes much to Major Ginter, for to him alone is due the present marked improvements in and around the city.

---

THE Clinical Society of St. James Dispensary, Savannah, is engaged in studying the coast malarias, and intends to devote eight or ten meetings to its discussion. We have arranged for reports of these meetings, the first of which will be found in this issue. The next meeting will discuss the clinical types of malaria, and subsequent meetings will take up the treatment of each type, and the preservation of man in malarious countries. The Society expects ultimately to have these papers and discussions published under one cover.

---

A JOINT resolution was recently adopted in the Georgia Legislature providing that all quarantine matters be entrusted to the United States Marine Hospital Service, and memorializing Congress to embody this resolution in a national quarantine law. The Governor of the State signified his disapproval of it by refusing to append his signature, on the plea that such action was contrary to the doctrine of State rights. Comment is unnecessary.—*Med. News.*

---

DO LABORATORY researches allow us to hope for the early discovery of a specific remedy for tuberculosis? Dr. Paul Gibier, of the New York Pasteur Institute, believes that they do. He is now experimenting with the "liquid of a culture of tuberculosis and anthrax," and he is able, at least, to report the cure of one inoculated tuberculous guinea-pig by this means (Bulletin, Pasteur Institute, December). "It seems to me that even in subjects most sensitive to the bacillus tuberculosis the development of this germ will, to a certainty, be eventually arrested. Hence I think we may venture to hope, through the medium of the experimental method, for the speedy advent of a therapeutic agent capable of producing this result in a ready and effective manner."

---

ACCORDING to announcement the *Philadelphia Medical Journal* made its appearance the first of January, and the three numbers that have been issued thus far have made a most favorable impression. Under the editorship of Dr. Gould its voice will give forth no uncertain sound upon topics that concern the welfare of the profession.

While we have been very much pleased with these numbers of the *Journal*, we beg to question the good judgment in placing the original articles at the last of contents. The order of the contents of a medical journal may have become so stereotyped that a greater or less departure from it may be acceptable, but this journal, in forsaking the old landmarks, has shown an excess of zeal in this particular which appears to us quite unnecessary. *The Philadelphia Medical Journal* will be conducted in the interest of the medical profession, and will not be owned by any publishing house or college. This is as it should be. We congratulate the *Journal* on its start, and we know it will score a great success.

---

WE refrain from boring our readers with any matter which may be in the least personal, but we may be excused for saying that the management of and keeping a medical journal going is no small job. Not only does it take money, time and a big lot of hard work, but a superabundance of grit and patience to move smoothly with a class of doctors who never pay their subscription. A doctor who will say: "O yes, we must have a medical journal—a State journal—every doctor should contribute to its support, and take a personal pride in the affair," and at the same time will not pay his subscription, or neglects to pay it, does not fully appreciate the serious aspect of the situation.

A man in New York rented a little corner, bought a small stove and other necessary implements, cooked and sold pies for a living. One day a silk-hat-kid-glove gentleman chanced to pass by and the pie vendor asked him to purchase a pie. The gentleman very politely stopped, talked very encouragingly and said he had often thought of the hard time the pie-sellers had; they worked very hard, lived hard, and he sympathized very, very much with all of them. The pie-seller, growing a little impatient, said: "D—— your sympathy! Buy a pie!"—*Alabama Med. and Surg. Age.*

---

MR. W. B. SAUNDERS, of Philadelphia, the well-known medical publisher, announces the following works in preparation for early publication: *American Text-Book of Diseases of the Eye, Ear, Nose and Throat,* by G. E. de Schwernitz, M.D., and Alexander Randall, M.D., of Philadelphia; *American Text-Book of Pathology,* by John Guiteras, M.D., and David Riesman, M.D., of Philadelphia; *American Text-Book of Legal Medicine and Toxicology,* by Frederick Peterson, M.D., of New York, and W. S. Haines, M.D., of Chicago; *Manual of Pathology,* by Alfred Stengel, M.D., of Philadelphia; *Nervous and Mental Diseases,* by Archibald Church, M.D., of Chicago, and Frederick Peterson, M.D., of New York; *Text-Book of Embryology,* by J. C. Heisler, M.D., of Philadelphia; *Diseases of the Nose and Throat,* by D. Braden Kyle, M.D., of Philadelphia; *Text-Book of Obstetrics,* by Bartin C. Hirst, of Philadelphia; *American Text Book of Nursing,* edited by Roberta West, of Philadelphia. Arrangements have also been completed for the publication of an English edition of the famous *Lehmann Medicinische Handatlanten.* Six or seven of these handsome atlases will be issued within the next three months, and others will probably follow. The *American Text-Book of Genito-Urinary and Skin Diseases,* by L. Balton Bangs, M.D., and W. A. Hardaway, M.D., the *Year Book of Medicine for 1898,* and Moore's *Orthopedic Surgery* are now ready.

---

DR. WILLIAM A. LOVE, of Atlanta, died January 22d, 1898. Dr. Love was one of the oldest and best known physicians in Georgia. He was born in Camden, South Carolina, in 1824, and when wenty years of age he entered the medical department of the University of Pennsylvania, from which he graduated in 1846. While a student in the university he also found time to devote to special work in the Obstetrical Institute under the instruction of Dr. Joseph Warrington, and in the Blockley and other hospitals in Philadelphia. In July, 1846, Dr. Love came to Georgia and located at Locust Grove, where he and Alexander Stephens soon became warm friends. He was elected Superintendent of the Georgia Asylum for the Deaf and Dumb at Cave Spring in 1850, a place which he filled with great success for eight years. In 1858 he moved to Albany, where he "soon established himself as a physician, surgeon, and gynecologist, and was regarded as the leading practitioner of that section." From Albany he went as volunteer in defense of the Confederate States, and here he served until the last hour of that eventful struggle, either in the field or in the hospitals. After the war Dr. Love removed to Atlanta and applied himself almost exclusively to the practice of gynecology and diseases of children, and at the same time he was elected professor of physiology in the Atlanta Medical College. Hundreds of students who have enjoyed his teaching for the last quarter of a century will testify to the ability with which he discharged the duties of this chair. Since 1893 he has been senior professor and president of the faculty of the Atlanta Medical College. Dr. Love was a member of the Georgia Medical Association and of the American Medical Association, and was vice-president of the physiological section of the International Medical Congress at Washington in 1887. He was a devoted Mason, and in national Masonic meetings he has been honored with important official positions. He was one of the "doctors of the old school," and, like MacLure, the hero of Drumtochty, an "honour tae oor profession."

His remains were buried with Masonic rites in Oakland cemetery January 24th.

